# Supplementary material for: Uncovering the environmental conditions required for Phyllachora maydis infection and tar spot development on corn in the United States for use as predictive models for future epidemics
Source: Sci Rep. 2023 Oct 10;13:17064. doi: 10.1038/s41598-023-44338-6 (PMC10564858; doi:10.1038/s41598-023-44338-6)
Supplement: Supplementary file 4 — Supplementary Legends. [file 41598_2023_44338_MOESM4_ESM.docx]

## **Supplementary Material**

Supplementary Table 1. Pearson correlation coefficient and significance p-value for 30-day moving average, 21-day moving average and 14-day moving average.

Supplementary Table 2. Weather parameters in logistic regression models for 30-day moving averages, 21-day moving averages, 14-day moving averages, and combined moving averages.

Supplementary Table 3. All information for small-plot trials and commercial fields planted in 2018 to 2022 in the following states: Illinois, Iowa, Indiana, Kentucky, Michigan, Missouri, Ohio, and Wisconsin in the United States.
